# Identification of a residue crucial for the angiostatic activity of human mini tryptophanyl-tRNA synthetase by focusing on its molecular evolution

**DOI:** 10.1038/srep24750

**Published:** 2016-04-20

**Authors:** Terumasa Nakamoto, Miki Miyanokoshi, Tomoaki Tanaka, Keisuke Wakasugi

**Affiliations:** 1 Department of Life Sciences, Graduate School of Arts and Sciences, The University of Tokyo, 3-8-1 Komaba, Meguro-ku, Tokyo 153-8902, Japan

## Abstract

Human tryptophanyl-tRNA synthetase (TrpRS) exists in two forms: a full-length TrpRS and a mini TrpRS. We previously found that human mini, but not full-length, TrpRS is an angiostatic factor. Moreover, it was shown that the interaction between mini TrpRS and the extracellular domain of vascular endothelial (VE)-cadherin is crucial for its angiostatic activity. However, the molecular mechanism of the angiostatic activity of human mini TrpRS is only partly understood. In the present study, we investigated the effects of truncated (mini) form of TrpRS proteins from human, bovine, or zebrafish on vascular endothelial growth factor (VEGF)-stimulated chemotaxis of human umbilical vein endothelial cells (HUVECs). We show that both human and bovine mini TrpRSs inhibited VEGF-induced endothelial migration, whereas zebrafish mini TrpRS did not. Next, to identify residues crucial for the angiostatic activity of human mini TrpRS, we prepared several site-directed mutants based on amino acid sequence alignments among TrpRSs from various species and demonstrated that a human mini K153Q TrpRS mutant cannot inhibit VEGF-stimulated HUVEC migration and cannot bind to the extracellular domain of VE-cadherin. Taken together, we conclude that the Lys153 residue of human mini TrpRS is a VE-cadherin binding site and is therefore crucial for its angiostatic activity.

Aminoacyl-tRNA synthetases catalyze the first step of protein synthesis, which comprises the aminoacylation of their cognate tRNAs^1^. Noncanonical functions distinct from aminoacylation have been reported, such as the cell-signaling functions of human tryptophanyl-tRNA synthetase (TrpRS) and tyrosyl-tRNA synthetase (TyrRS) in pathways connected to the immune system or angiogenesis^2^,^3^,^4^,^5^,^6^,^7^.

Vertebrate TrpRSs have an NH_2_-terminal appended domain. In normal cells, human TrpRS exists in two forms: the major full-length protein form and a less abundant mini TrpRS, in which the extra NH_2_-terminal domain is deleted due to alternative splicing of the pre-mRNA such that Met48 becomes the NH_2_-terminal residue^8^,^9^ (Fig. 1). We previously found that human mini, but not full-length, TrpRS functions as an angiostatic factor^5^. Full-length TrpRS (a.a. 1–471) is cleaved by elastase to produce T1 TrpRS (a.a. 71–471) and T2 TrpRS (a.a. 94–471), which also act as angiostatic factors^5^,^10^. Whereas full-length, mini and T1 TrpRSs retain aminoacylation activity, T2 TrpRS is inactive for aminoacylation^10^.

Vascular endothelial (VE)-cadherin was identified as a target for the angiostatic activity of truncated (mini and T2) TrpRSs^11^,^12^,^13^,^14^. VE-cadherin belongs to the cadherin superfamily of cell-cell adhesion molecules and plays a key role in vascular endothelial growth factor (VEGF)-mediated endothelial survival, endothelial barrier function, and angiogenesis^15^. Functional blocking monoclonal antibodies against VE-cadherin inhibited angiogenesis^16^. VE-cadherin consists of an extracellular domain, which comprises five extracellular cadherin repeats (EC1-EC5), a transmembrane domain, and a COOH-terminal cytoplasmic domain responsible for interacting with catenin^15^. VEGF binds to its receptor, vascular endothelial growth factor receptor 2 (VEGFR2), and a multicomponent complex comprising VE-cadherin, β-catenin, phosphoinositide 3 kinase and VEGFR2 is formed, which activates Akt and promotes endothelial survival^12^,^17^. It has been found that human truncated (mini and T2) TrpRSs bind to the extracellular domain of VE-cadherin and these TrpRSs have been suggested to target VE-cadherin and block VEGF-mediated association of VE-cadherin with VEGFR2 in addition to the transmission of the endothelial survival signal by VEGF to Akt^11^,^12^,^13^,^14^.

The expression of human full-length and mini TrpRSs is increased following stimulation of human cells by interferon-γ (IFN-γ)^18^,^19^,^20^,^21^,^22^,^23^,^24^. Human TrpRS is the only aminoacyl-tRNA synthetase whose expression is induced by IFN-γ. Moreover, we recently showed that the expression of TrpRS is also enhanced by exposure of mouse cells to IFN-γ^25^. It should also be noted that bovine TrpRS is highly expressed in the pancreas and is secreted into the pancreatic juice^26^,^27^,^28^,^29^,^30^, thus resulting in the production of truncated TrpRSs in which the extra NH_2_-terminal domain is deleted by proteolysis^26^.

We have been investigating the aminoacylation activity of TrpRSs from several species^25^,^31^,^32^. For example, we demonstrated that Zn^2+^-depleted human TrpRS is enzymatically inactive and that binding of Zn^2+^ or heme to human TrpRS stimulates its aminoacylation activity^31^,^32^. Bovine and mouse TrpRSs were found to be constitutively active regardless of the presence of Zn^2+^ or heme^31^,^32^. These results provide evidence for species-specific regulation of TrpRS aminoacylation activity.

In the present study, we created truncated forms of bovine and zebrafish TrpRSs, which are similar in size to the human mini TrpRS, based on sequence alignment analysis and designated these truncated TrpRSs as the “mini” TrpRSs (Fig. 1). We first investigated and compared the angiostatic activities of full-length and mini TrpRSs of human, bovine, and zebrafish TrpRSs, and arabidopsis full-length TrpRS. As shown in Fig. 1, arabidopsis full-length TrpRS does not have the NH_2_-terminal appended domain. Next, to identify residues crucial for angiostatic activity of human mini TrpRS, we aligned the sequences of the human, bovine, and zebrafish TrpRS proteins, prepared site-directed mini TrpRS mutants, and investigated their angiostatic activities. Because it has been reported that human T2 and mini TrpRSs bind to VE-cadherin and that the interaction of truncated TrpRSs with VE-cadherin is crucial for their angiostatic activities^11^,^12^,^13^,^14^, we next investigated the binding of mini wild-type (WT) TrpRS or mutant to VE-cadherin by immunoprecipitation assays. Moreover, we propose a model of the complex between human mini TrpRS and VE-cadherin based on the aminoacylation activities of TrpRS mutants.

## Results

### Comparison of the inhibition of VEGF-dependent chemotaxis of human umbilical vein endothelial cells (HUVECs) by the various TrpRSs

**Figure 2:**
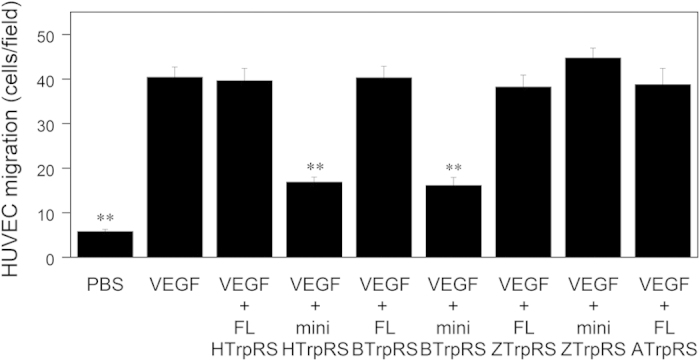
Effects of human, bovine, zebrafish and arabidopsis TrpRSs on VEGF-induced endothelial migration. VEGF (0.5 nM) and full-length (FL) or mini TrpRS (500 nM) were used. Migrating cells were counted in four random fields (×100 total magnification) per insert and were averaged. All data are expressed as means ± SEM from at least four independent experiments. Data were analyzed by one-way ANOVA followed by Tukey-Kramer post hoc tests. **P < 0.01 versus the VEGF control.

Human, bovine, zebrafish and arabidopsis TrpRSs were purified following expression in *E. coli* (Supplementary Fig. S1). We compared the angiostatic activities of full-length and mini forms of human, bovine, and zebrafish TrpRSs, and arabidopsis TrpRS, as assessed by the ability to inhibit HUVEC migration. As shown in Fig. 2, VEGF alone induced HUVEC chemotaxis. Next, we exposed VEGF-stimulated HUVEC cells to human mini TrpRS, and observed that it inhibited VEGF-induced migration (Fig. 2). In contrast, no inhibition of chemotaxis was observed with human full-length TrpRS (Fig. 2). These results are consistent with our previous results^5^. Moreover, we observed that bovine mini TrpRS inhibited VEGF-stimulated HUVEC chemotaxis to a similar extent as human mini TrpRS, whereas zebrafish mini TrpRS and arabidopsis TrpRS did not (Fig. 2). In addition, the bovine full-length TrpRS and zebrafish full-length TrpRS had no effect on VEGF-stimulated HUVEC chemotaxis (Fig. 2). These results show that bovine, but not zebrafish, TrpRS can function as an angiostatic factor after removal of the extra domain from the full-length TrpRS.

### Identification of an amino acid residue of mini TrpRS crucial for the inhibition of VEGF-induced HUVEC chemotaxis

**Figure 3:**
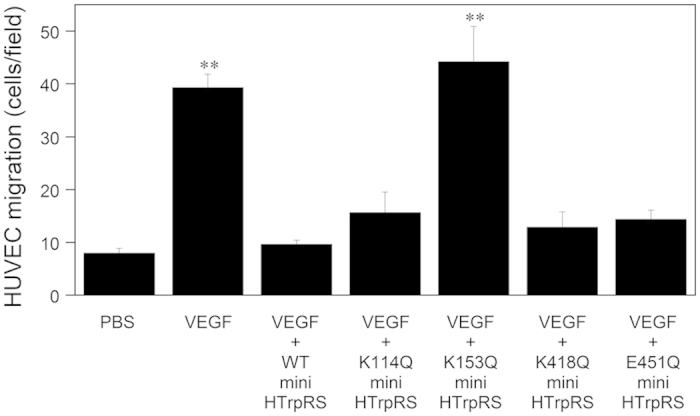
Effects of human mini WT, K114Q, K153Q, K418Q, and E451Q TrpRSs on VEGF-induced endothelial migration. VEGF (0.5 nM) and mini TrpRS (500 nM) were used. Migrating cells were counted in four random fields (×100 total magnification) per insert and were averaged. All data are expressed as means ± SEM from at least four independent experiments. Data were analyzed by one-way ANOVA followed by Tukey-Kramer post hoc tests. **P < 0.01 compared to the VEGF plus human mini WT TrpRS.

**Figure 4:**
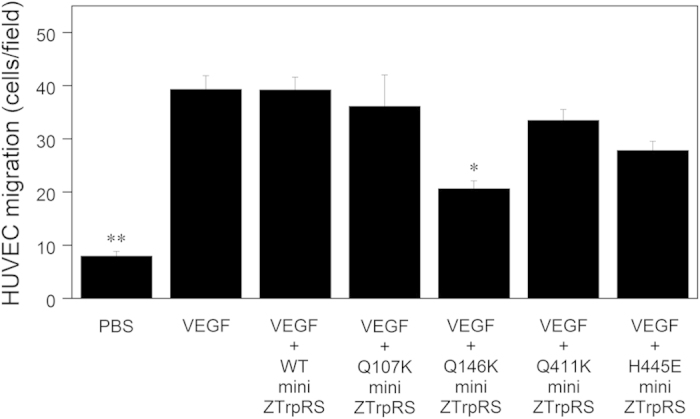
Effects of zebrafish mini WT, Q107K, Q146K, Q411K, and H445E TrpRSs on VEGF-induced endothelial migration. VEGF (0.5 nM) and mini TrpRS (500 nM) were used. Migrating cells were counted in four random fields (×100 total magnification) per insert and were averaged. All data are expressed as means ± SEM from at least four independent experiments. Data were analyzed by one-way ANOVA followed by Tukey-Kramer post hoc tests. *P < 0.05, **P < 0.01 compared to the VEGF plus zebrafish mini WT TrpRS.

Next, to identify crucial residues for the angiostatic activity of human mini TrpRS, we performed a sequence alignment between mammalian and fish TrpRS proteins and pinpointed key differences between mammalian and fish TrpRS sequences, with a particular focus on exposed residues with positive or negative charges (Supplementary Fig. S2). We identified Lys114, Lys153, Lys418, and Glu451 of human TrpRS, which correspond to Gln107, Gln146, Gln411, and His445 of zebrafish TrpRS, respectively, as potential candidate residues, and prepared the site-directed human mini TrpRS mutants, K114Q, K153Q, K418Q, and E451Q, and the zebrafish mini TrpRS mutants, Q107K, Q146K, Q411K, and H445E, with numbering based on the residue numbers of full-length TrpRS. We examined the effects of TrpRS mutants on VEGF-induced cell migration. As shown in Fig. 3, we observed that the human mini K153Q TrpRS mutant did not inhibit VEGF-stimulated HUVEC chemotaxis, whereas the human mini K114Q, K418Q, and E451Q TrpRS mutants inhibited VEGF-stimulated HUVEC migration as did the human mini WT TrpRS. In addition, we show that the zebrafish mini Q146K TrpRS mutant, in which Gln146 (corresponding to Lys153 of human TrpRS) was converted to Lys, significantly inhibited VEGF-stimulated HUVEC migration (Fig. 4). Taken together, these data suggest that Lys153 of human mini TrpRS is crucial for its angiostatic activity.

### Binding of TrpRSs to VE-cadherin

**Figure 5:**
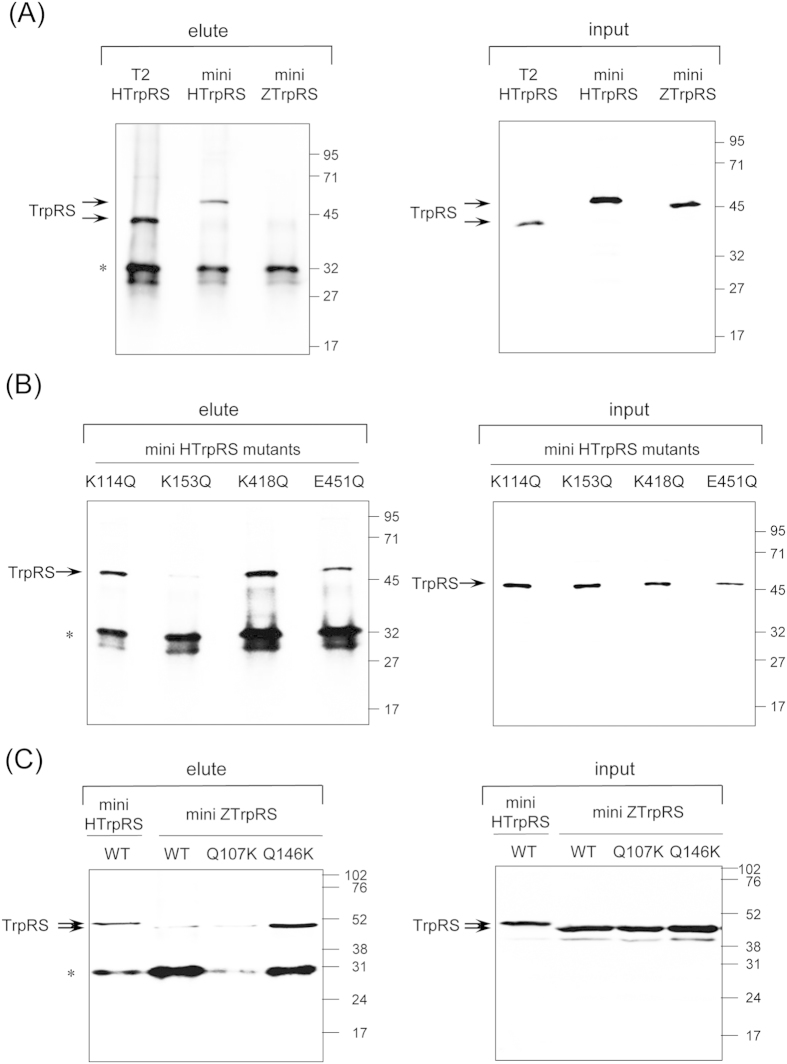
Analysis of the interaction of TrpRS with human VE-cadherin by co-immunoprecipitation assays. (**A**) VE-cadherin binding assay of human T2, mini WT TrpRS, and zebrafish mini WT TrpRS. (**B**) VE-cadherin binding assay of human mini K114Q, K153Q, K418Q, and E451Q TrpRS mutants. (**C**) VE-cadherin binding assay of human mini WT TrpRS, zebrafish mini WT TrpRS, zebrafish mini Q107K and Q146K TrpRS mutants. Rabbit polyclonal antibodies against TrpRS were used for Western blot analyses of panels **A** and **B**. For panel **C**, mouse monoclonal antibody against six histidine residues was used. Molecular size markers (in kilodaltons) are shown at the right. A band corresponding to TrpRS or protein G derived from protein G plus-agarose is marked with an arrow or asterisk, respectively.

Immunoprecipitation experiments using a recombinant human VE-cadherin Fc chimera, comprising the extracellular domain (EC1-EC5) of human VE-cadherin fused to the Fc domain of human IgG, revealed that human mini WT TrpRS binds to the extracellular domain of VE-cadherin, as does the human T2 TrpRS (Fig. 5A). These data are consistent with previous findings^13^,^14^. In contrast, the zebrafish mini WT TrpRS did not bind to VE-cadherin (Fig. 5A). Next we performed immunoprecipitation experiments using the human mini TrpRS mutants to identify the residues of human mini TrpRS crucial for binding to VE-cadherin. As shown in Fig. 5B, human mini K114Q, K418Q, and E451Q TrpRS mutants bound to VE-cadherin, whereas the human mini K153Q TrpRS mutant did not. These data suggest that the Lys153 residue of human mini TrpRS is crucial for the interaction with VE-cadherin. Furthermore, the zebrafish mini Q146K TrpRS mutant interacted with VE-cadherin significantly as did human mini WT TrpRS (Fig. 5C). Taken together, we conclude that Lys153 of human mini TrpRS is a VE-cadherin binding site.

### Aminoacylation activities of TrpRSs

**Figure 6:**
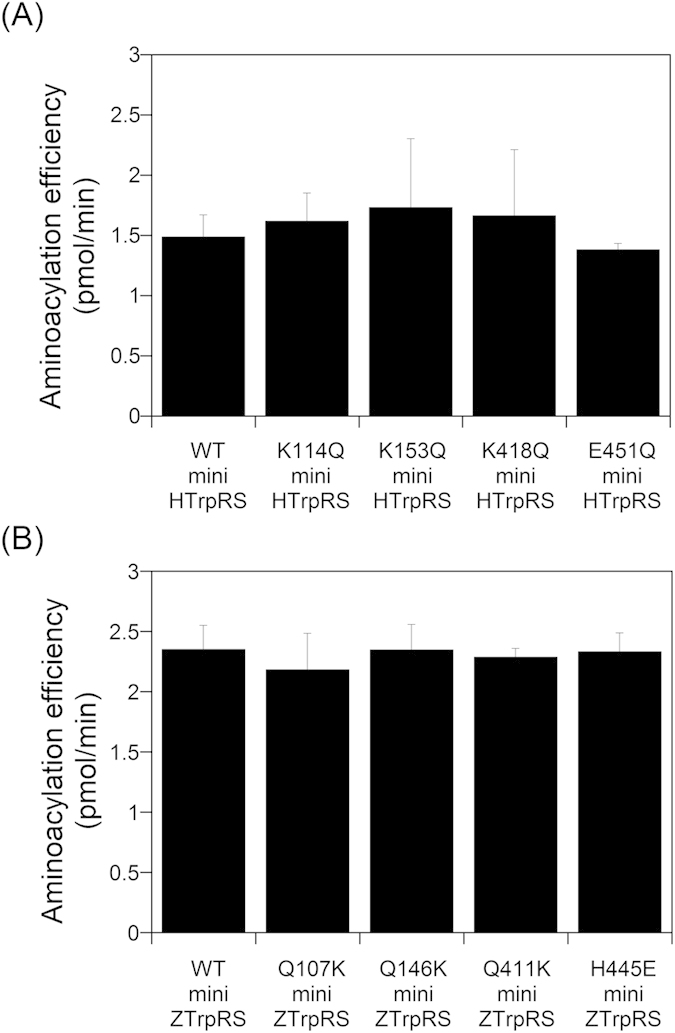
Aminoacylation activity of human and zebrafish TrpRSs toward yeast tRNA^Trp^. Aminoacylation efficiencies were determined from initial rates and calculated as pmol/min of aminoacylated tRNA^Trp^ synthesized during a 1 min incubation. The assays included 200 nM TrpRS and 500 μM yeast tRNA. Values represent the means ± standard deviation from four experiments. (**A**) Aminoacylation activity of human mini WT, K114Q, K153Q, K418Q, and E451Q TrpRSs. (**B**) Aminoacylation activity of zebrafish mini WT, Q107K, Q146K, Q411K, and H445E TrpRSs.

Because it has been previously reported that the NH_2_-terminal Trp2 and Trp4 residues of VE-cadherin are docked into the Trp- and adenosine-binding pockets of human TrpRS^13^, we next investigated the aminoacylation activity of the various TrpRSs. Since previous data showed that eukaryotic TrpRSs can aminoacylate yeast tRNA^Trp ^31,32,33,34,35, we investigated the aminoacylation of yeast tRNA^Trp^ by the recombinant TrpRSs. As shown in Fig. 6A, we observed that the human mini K153Q TrpRS mutant exhibited similar aminoacylation activity as the human mini WT TrpRS, implying that the K153Q mutation has no effect upon the tertiary structure within the Trp- and adenosine-binding pockets. Moreover, the aminoacylation activity of the zebrafish mini Q146K TrpRS mutant was also almost the same as that of the zebrafish mini WT TrpRS (Fig. 6B). Taken together, we conclude that Lys153 of human mini TrpRS is crucial for its angiostatic activity but not for its aminoacylation activity.

## Discussion

**Figure 7:**
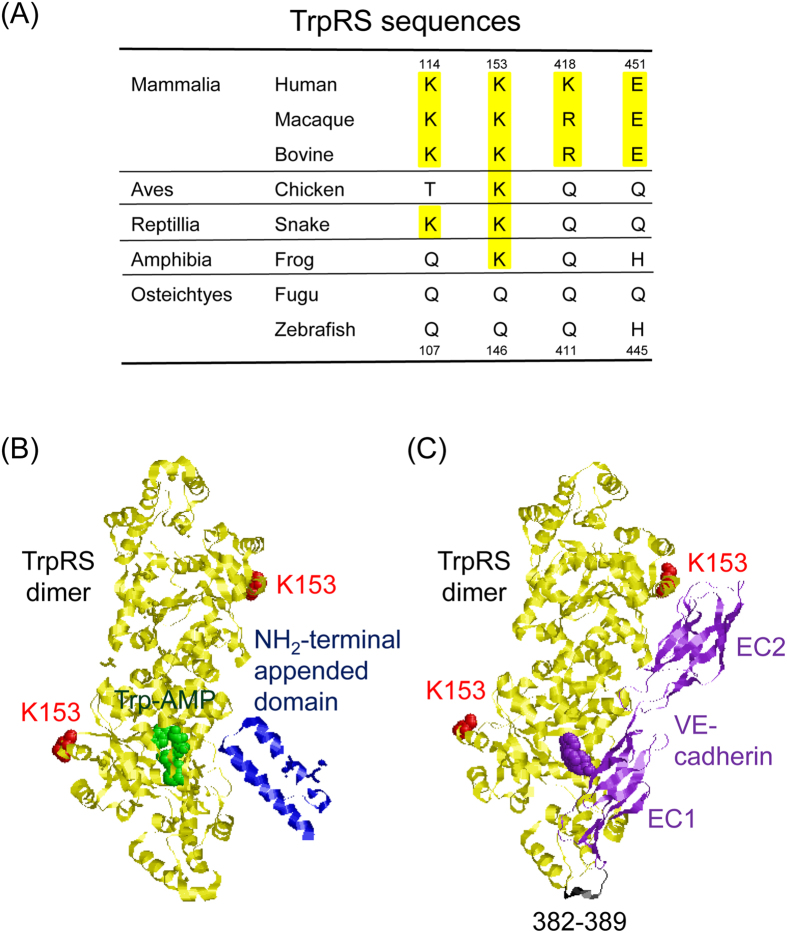
A residue of human TrpRS crucial for angiostatic activity. (**A**) Comparison of amino acid residues at the positions tested for angiostatic activity in this study among mammalian, bird, reptilian, amphibian, and fish TrpRS proteins. Multiple sequence alignment was performed by Clustal W with manual adjustments. Conserved crucial acidic (Glu or Asp) and basic (Arg or Lys) residues are highlighted in yellow. (**B**) Tertiary structural conformation of Lys153 and the NH_2_-terminal appended domain of human full-length TrpRS, and tryptophanyl-adenylate (Trp-AMP) bound to human full-length TrpRS (Protein Data Bank code: 1R6T). Lys153, NH_2_-terminal appended domain, and Trp-AMP are indicated in red, blue and green, respectively. (**C**) A molecular docking model of the complex between human mini TrpRS (Protein Data Bank code: 1ULH) and EC1-EC2 of VE-cadherin (Protein Data Bank code: 3PPE). Lys153 and 382–389 resides in human mini TrpRS are indicated in red and black, respectively. Human mini TrpRS and EC1-EC2 of VE-cadherin are highlighted in yellow and purple, respectively. NH_2_-terminal Trp2 and Trp4 residues of VE-cadherin are represented by purple space-filling balls.

In the present study, we showed that bovine mini TrpRS inhibited VEGF-stimulated HUVEC chemotaxis to an extent similar to that of human mini TrpRS, whereas zebrafish mini or arabidopsis full-length TrpRS did not. We further demonstrated that Lys153 of human mini TrpRS is crucial for its angiostatic activity but not for its aminoacylation activity. Lys153 is located in the eukaryote-specific patch, which extends from Glu82 to Lys154^36^. As shown in Fig. 7A, Lys153 is conserved among mammalia, aves, reptillia, and amphibia, but not among osteichtes, implying that the mini TrpRSs from mammalia, aves, reptillia, and amphibia may act as angiostatic factors. Further studies are necessary to investigate protein-protein interactions between TrpRS and VE-cadherin from various species and to examine the angiostatic activities of TrpRSs by using endothelial cells from various species.

The catalytic domain of TrpRS has a tertiary and quaternary structure similar to that of tyrosyl-tRNA synthetase (TyrRS)^37^,^38^,^39^,^40^. Human TyrRS has an extra domain at the COOH-terminus compared to lower eukaryotic TyrRSs. We previously found that human TyrRS can be proteolyzed into two fragments with distinct cytokine activities, i.e. the NH_2_-terminal catalytic domain (mini TyrRS) and the COOH-terminal extra appended domain^2^,^3^. We further reported that human mini TyrRS acts as an angiogenic factor^4^. Human mini TrpRS and mini TyrRS belong to a family of regulators of angiogenesis and have opposing cell-signaling activities. We previously found that the Glu-Leu-Arg (ELR) motif of human mini TyrRS is crucial for its angiogenic activity and receptor binding^2^,^3^. All vertebrate TyrRSs have an extra COOH-terminal appended domain and the ELR motif is conserved among TyrRSs from all vertebrates, such as mammalia, aves, reptillia, amphibia, and osteichtes. Therefore, we speculate that zebrafish mini TyrRS functions as an angiogenic factor, as does the human mini TyrRS. In the present study, we clarified that zebrafish mini TrpRS cannot act as an angiostatic factor, implying that the angiostatic function of TrpRS may be evolutionally more recent than angiogenic function of TyrRS.

In the present study, we demonstrated that Lys153 of human mini TrpRS is crucial for its angiostatic activity and its interaction with VE-cadherin. It has been previously reported that a human mini G161W TrpRS mutant, in which the Trp-binding pocket of TrpRS is blocked by the bulky indole side chain of a Trp residue introduced at position 161, exhibited substantially diminished binding to VE-cadherin compared to mini WT TrpRS^14^. It has also been proposed that the NH_2_-terminal Trp2 and Trp4 residues of EC1 of VE-cadherin are docked into the Trp- and adenosine-binding pockets of TrpRS^13^. The finding that the aminoacylation activity of the K153Q mutant is similar to that of WT TrpRS suggests that the K153 mutation does not affect the Trp- and adenosine-binding pockets, which bind to VE-cadherin. Taken together, we propose that Lys153 is a VE-cadherin-binding site.

Moreover, it has previously been reported that a human mini TrpRS mutant lacking residues 382–389 within the anticodon-binding domain lost its angiostatic activity^40^. Therefore, both Lys153 and residues 382–389 of mini TrpRS may play important roles in its angiostatic activity and its interaction with VE-cadherin. Based on this information, we created a molecular docking model of the complex between TrpRS and VE-cadherin. Since the Trp- and adenosine-binding pockets crucial for the interaction with VE-cadherin in human TrpRS can be covered by the NH_2_-terminal appended domain sterically (Fig. 7B,C), this model is consistent with previous experimental results that the existence of the NH_2_-terminal appended domain of human full-length TrpRS blocks the interaction between TrpRS and VE-cadherin^13^ and the present result that human TrpRS can function as an angiostatic factor after removal of the NH_2_-terminal appended domain from the full-length TrpRS. As shown in Fig. 7B,C, human TrpRS is a dimer of two identical subunits. In one subunit, Lys153 residue is located at the opposite site of the binding surface with VE-cadherin (Fig. 7C). However, Lys153 residue in the other subunit of human TrpRS may interact with EC2 of VE-cadherin (Fig. 7C). Further studies are necessary to clarify the detailed protein-protein interactions between human mini TrpRS and VE-cadherin.

## Methods

### Chemicals

Brewer’s yeast tRNA was purchased from Roche Diagnostics (Basel, Switzerland). Human vascular endothelial growth factor-165 (VEGF) from Pepro Tech Inc. (RockyHill, NJ) was used.

### Preparation of proteins

Human, bovine and zebrafish full-length TrpRS cDNA clones were purchased from Open Biosystems (Huntsville, AL). Arabidopsis full-length TrpRS cDNA clone was obtained from RIKEN BioResource Center (Ibaraki, Japan). A cDNA fragment from human, bovine, zebrafish, or arabidopsis TrpRS was separately cloned into the pET20b (Novagen, Madison, WI) expression vector so as to generate a gene product with a COOH-terminal tag of six histidine residues^31^,^32^. A QuikChange^TM^ site-directed mutagenesis system (Stratagene, La Jolla, CA) was used to introduce substitutions at specific sites. The final constructs were confirmed by DNA sequencing (FASMAC Co., Ltd., DNA sequencing services, Atsugi, Japan) to ensure no mistakes had been introduced during amplification. The expression constructs were introduced into *E. coli* BL21(DE3) (Novagen). The cells were grown at 37 °C to an OD_600_ of approximately 0.8 and then heterologous gene expression was induced by the addition of 0.4 mM isopropyl β-D-thiogalactopyranoside. Cells were harvested 4 h after induction. According to the procedures described by Novagen, the recombinant histidine-tagged proteins were purified on a nickel affinity column (His•Bind^®^ resin; Novagen) from the supernatant of lysed cells. Endotoxin was removed from the protein solutions by phase separation using Triton X-114^41^,^42^ and was determined to be <0.5 endotoxin units/ml by a *Limulus* amebocyte lysate gel-clot assay (E-TOXATE kit; Sigma-Aldrich, St. Louis, MO). Protein concentration was determined by using Bradford protein assay reagent (Bio-Rad Laboratories Inc., Hercules, CA) and bovine serum albumin (BSA) (Sigma-Aldrich) as the standard.

### Cell line

Human umbilical vein endothelial cells (HUVECs) (HUVEC-2; BD Biosciences, San Jose, CA) were maintained in Clonetics EGM^®^-2 BulletKit^®^ medium (Takara Bio Inc., Otsu, Japan) in an atmosphere of 5% CO_2_ in air at 37 °C according to the instructions of the supplier. Cells were maintained in logarithmic growth phase by routine passage every 2–3 days.

### HUVEC chemotaxis assays

Cell migration was measured using 24-well culture plates (Iwaki, Tokyo, Japan) and modified Boyden chambers (Chemotaxicell; Kurabo, Osaka, Japan) with polycarbonate membranes (8.0 μm pore size) as described previously^4^,^5^. The wells were coated with 25 μg/ml human fibronectin (Sigma-Aldrich) in phosphate-buffered saline (PBS) overnight and were allowed to air dry. At first, HUVECs were incubated in serum-free Dulbecco’s modified Eagle’s medium (DMEM) (Invitrogen, Carlsbad, CA) containing 2 mM glutamine and 0.1% BSA (Sigma-Aldrich) under conditions of serum starvation for 4 h. HUVECs were then suspended in DMEM (Invitrogen) containing 2 mM glutamine and 0.1% BSA (Sigma-Aldrich) and 2 × 10^5^ cells/well were added to the upper chamber. VEGF (0.5 nM) was placed in the lower chamber. Inhibition assays were conducted with the addition of TrpRS (500 nM) to both the upper and lower chambers. HUVECs were suspended in media with TrpRS for 30 min before placement in the upper chamber. Then, the cells were allowed to migrate for 6 hours at 37 °C in a 5% CO_2_ incubator. After incubation, non-migrant cells were removed from the upper face of the membrane with a cotton swab and migrant cells, those attached to the lower face, were fixed in methanol and visualized with the Hemacolor^®^ staining kit (Merck, Darmstadt, Germany). HUVEC migration was quantified by counting the number of cells in four random fields (×100 total magnification) per insert.

### Immunoprecipitation

Recombinat human VE-cadherin Fc chimera (R & D Systems, Minneapolis, MN) (52 nM), consisting of the extracellular domain (EC1-EC5) of human VE-cadherin fused to the Fc domain of human IgG, were incubated with TrpRS (120 nM) in buffer A (50 mM Tris-HCl (pH 7.5), 150 mM NaCl, 1% (w/v) Triton X-100, and 5 mM CaCl_2_) for 1 h and mixed with protein G plus-agarose (Santa Cruz Biotechnology, Santa Cruz, CA). Then, the mixture was incubated for 1 h. After washing with buffer A three times, protein G-bound proteins were eluted with 5% (w/v) SDS and 10 mM DTT and were analyzed by Western blot analyses.

### Western blot analyses

The samples were separated through 12% polyacrylamide-SDS gels. Proteins were transferred onto Hybond-P PVDF membranes (GE Healthcare Biosciences, Piscataway, NJ), which were then blocked with PBS containing 5% skim milk (Wako Pure Chemical Industries, Osaka, Japan). The membranes were incubated for 1 h in PBS containing rabbit polyclonal antibodies against human mini TrpRS, which were prepared by custom polyclonal antibody production services (Takara Bio Inc.), or the mouse monoclonal antibody directed against six histidine residues (Invitrogen). After washing, the membranes were incubated with an HRP-linked F(ab’)_2_ fragment of donkey anti-rabbit IgG or an HRP-linked whole antibody of sheep anti-mouse IgG (GE Healthcare Biosciences) for 1 h, respectively. The membranes were again washed three times with the buffer, and the proteins were visualized using ECL^tm^ western blotting detection reagents (GE Healthcare Biosciences). Chemiluminescent signals were detected using a LAS-4000 mini luminescent image analyzer (GE Healthcare Biosciences).

### Aminoacylation assays

Aminoacylation activity was assayed in buffer containing the following: 150 mM mM Tris-HCl (pH 7.5), 150 mM KCl, 10 mM MgCl_2_, 4 mM ATP, and 21 μM tryptophan (Trp) [1 μM [^3^H]Trp (PerkinElmer, Waltham, MA)]. The reactions were initiated by adding the purified samples (200 nM) to the buffer that included Brewer’s yeast tRNA (500 μM). Reaction samples were removed and spotted onto Whatman 3MM paper filters. After 0, 2, 4, and 6 min, the filter discs were added to cold 5% trichloroacetic acid that included 2 mM Trp. The filters were washed three times in cold 5% trichloroacetic acid and 2 mM Trp, once in ethanol and dried. The washed filters were then counted in a liquid scintillation counter (LS6500; Beckman Coulter, Fullerton, CA).

## Additional Information

**How to cite this article**: Nakamoto, T. *et al.* Identification of a residue crucial for the angiostatic activity of human mini tryptophanyl-tRNA synthetase by focusing on its molecular evolution. *Sci. Rep.* **6**, 24750; doi: 10.1038/srep24750 (2016).

## Supplementary Material

Supplementary Information

## Figures and Tables

**Figure 1:**
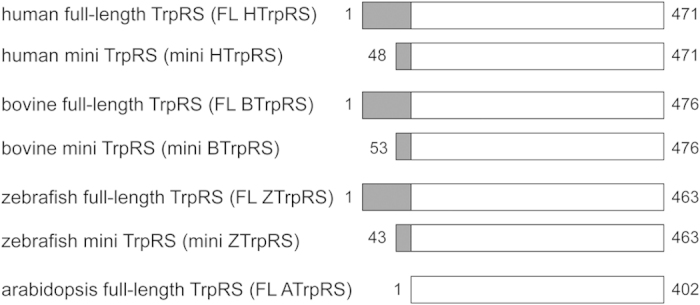
Schematic representation of human, bovine, zebrafish and arabidopsis TrpRS constructs used in this study. Sequence alignments of full-length (FL) and mini TrpRS proteins are depicted schematically. Numbers on the left and right correspond to the NH_2_- and COOH-terminal residues, respectively. The open boxes indicate the core catalytic domain conserved among eukaryotic TrpRSs and the shaded boxes represent the NH_2_-terminal appended domains specific to vertebrate TrpRSs.
