# Supplementary material for: Identification of a residue crucial for the angiostatic activity of human mini tryptophanyl-tRNA synthetase by focusing on its molecular evolution
Source: Sci Rep. 2016 Apr 20;6:24750. doi: 10.1038/srep24750 (PMC4837363; doi:10.1038/srep24750)
Supplement: Supplementary Information [file srep24750-s1.pdf]

Supplementary Figures

Identification of a residue crucial for the angiostatic activity of human mini tryptophanyl-tRNA synthetase by focusing on its molecular evolution

Terumasa Nakamoto, Miki Miyanokoshi, Tomoaki Tanaka, and Keisuke Wakasugi*

Department of Life Sciences, Graduate School of Arts and Sciences, The University of Tokyo, 3-8-1 Komaba, Meguro-ku, Tokyo 153-8902, Japan.

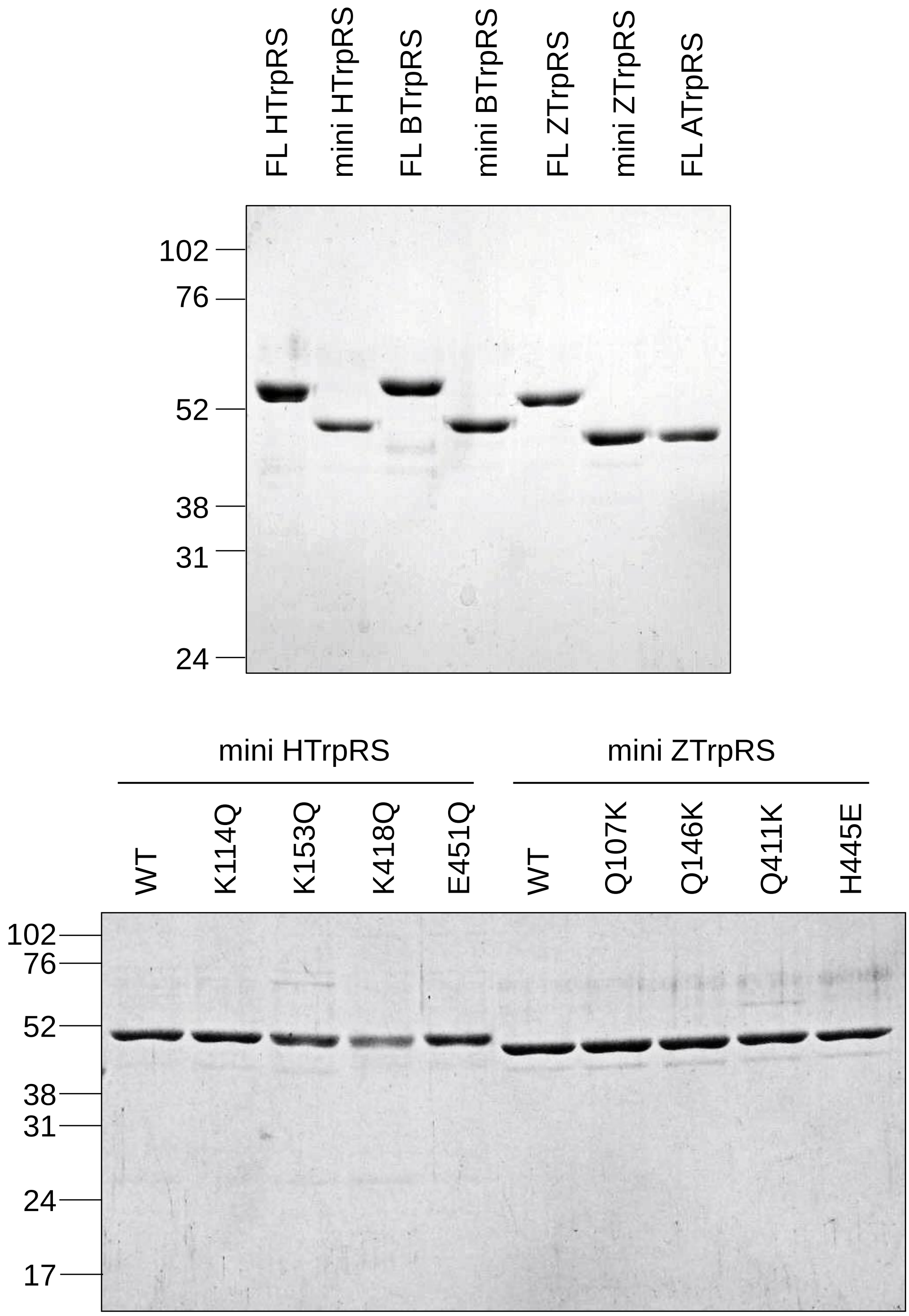

Supplementary Figure S1. SDS-polyacrylamide gel electrophoresis of human, bovine, zebrafish and arabidopsis TrpRS constructs used in this study. The samples were analyzed on a 12.0% SDS-polyacrylamide gel and stained with Coomassie Blue. Molecular size markers (in kilodaltons) are shown at the left.

```
                      1                                              → mini                                                    → T2
Human       -----MPNSEPASLLELFNSIATQGELVRSLKAGNASKDEIDSAVKMLVSLKMSYKAAAGEDYKADCPPGNPAPTSNHGPDATEAEEDFVDPWTVQTSSAKGIDYDKLIVRFGS
Macaque     -MANMPNSEPCASPLELFNSIATQGELVRSLKAGNASKDEIDSAVKMLLSLKMSYKAAMGEDYKADCPPGNPAPTSNHGPDAAEAEEDFVDPWTVQTSSAKGIDYDKLIVRFGS
Bovine      MADMSNGEQGCGSPLELFHSIAAQGELVRDLKARNAAKDEIDSAVKMLLSLKTSYKAATGEDYKVDCPPGDPAPESGEGLDATEADEDFVDPWTVQTSSAKGIDYDKLIVRFGS
Chicken     MADSPNCDLKSLSPLQLFEKVTEQGEKVRALKAGKAPKDEIDAAVRLLLSLKLSYKTTTGQDYQAGLPPKDHALINNG--TTKEEDEDLVDPWNVQTSNAKGVDYDKLIVRFGS
Snake       MADNPNGSFCSLKPLELFEQIAAQGENVRSLKSKKESKDEIDAAVKLLLSLKVTYKTATGQEYKADSPPTDFTPVSNS--PDNIEGDDFVDPWTVQTSNAKGVDYDKLIVRFGS
Frog        MSDSGTDASGSLIPMDLYDRVTAQGDKVRVLKSEKAPKEEIDVAVKLLLALKVDYKNVTGQDYKPGVPPADDMPTNSNGPSTTDNGDDFVDPWSVQTGSAKGVDYDKLIVRFGS
Fugu        -MADSPDFERTMSPLELYEKLTAQGDQVRALKTAKSDKAEIDAAVQLLLKMKLEYKQVTGQDYKAGSPPSENSAPFNGATADGSNDEDTVDPWNVSTTNAKGVDYDKLIVRFGS
Zebrafish   ----------MSSPLDLYECVTAQGETVRSIKAKKGSKEEVDAAVQVLLQMKLDYKRVTGQDYKAGCPPQDGVTPQDGAAP--QDGEDQVDPWSVSSSSAKGVDYEKLIVRFGS

                114                                  153
Human       SKIDKELINRIERATGQRPHHFLRRGIFFSHRDMNQVLDAYENKKPFYLYTGRGPSSEAMHVGHLIPFIFTKWLQDVFNVPLVIQMTDDEKYLWKDLTLDQAYSYAVENAKDII
Macaque     SKIDKELINRIERATGQRPHRFLRRGIFFSHRDMNQVLDAYENKKPFYLYTGRGPSSEAMHVGHLIPFIFTKWLQDVFNVPLVIQMTDDEKYLWKDLTLDQAYGYAVENAKDII
Bovine      SKIDKELVNRIERATGQRPHRFLRRGIFFSHRDMHQILDAYENKKPFYLYTGRGPSSEAMHVGHLIPFIFTKWLQDVFNVPLVIQMTDDEKYLWKDLTLDQAYGYAVENAKDII
Chicken     TKIDTDLINRIERATGQKPHRFLRRGIFFSHRDMDQILHAYENKKPFYLYTGRGPSSQAMHVGHLIPFLFTKWLQEAFDVPLVIQLTDDEKYLWKDMTTEKAYEYAKENARDII
Snake       SKIDKDLINRIERITGHKPHHFLRRGIFFSHRDMNQILDAHEKKKPFYLYTGRGPSSETMHVGHLIPFMFTKWLQETFNVPLVIQMTDDEKYLWKDLTIEKAYQYAIENIKDII
Frog        SKIDQELVDRIQRVTGQKPHHFLRRGIFFSHRDMHQVLDAYENKKPFYLYTGRGPSSEALHVGHLIPFIFTKWLQEVFNVPLVVQLTDDEKYLWKDLTLEKAYQYSIENAKDII
Fugu        SKIDQQLVDRIEKVSGQKPHRFLRRGIFFSHRDMHQVLDAYEKQKSFYLYTGRGPSSEAMHVGHLIPFIFTKWLQDVFDIPLVIQLTDDEKYLWKDLTIEECHRFAVENAKDII
Zebrafish   SKVDQELVDRISRLTGKTPHRFLRRGIFFSHRDMHQILDAFEQQKPFYLYTGRGPSSQAIHVGHLIPFIFTKWLQDVFDVPLVIQLTDDEKYLWKDLTLEECRRFTVENARDII

Human       ACGFDINKTFIFSDLDYMGMSSGFYKNVVKIQKHVTFNQVKGIFGFTDSDCIGKISFPAIQAAPSFSNSFPQIFRDRTDIQCLIPCAIDQDPYFRMTRDVAPRIGYPKPALLHS
Macaque     ACGFDINKTFIFSDLDYMGMSSGFYKNVVKIQKHVTFNQVKGIFGFTDSDSIGKISFPAIQAAPSFSNSFPQIFQDRTDIQCLIPCAIDQDPYFRMTRDVAPRIGYPKPALLHS
Bovine      ACGFDINKTFIFSDLDYMGMSPGFYKNVVKIQKHVTFNQVKGIFGFTDSDCIGKISFPAIQAAPSFSNSFPQIFRDRTDVQCLIPCAIDQDPYFRMTRDVAPRIGYPKPALLHS
Chicken     ACGFDVNKTFIFSDLDYLGSSTGFYKNIIKVQKHVTFNQVKGIFGFTDSDCIGKISFPAIQAAPSFSSSFPQIFNGKENIQCLIPCAIDQDPYFRMTRDVAPRIGYPKPALLHS
Snake       ACGFDINKTFIFSDFSYIGTSLGFYKNIVKVQKHVTFNQVKGIFGFTDSDCIGKISFPAIQAAPSFSSSFPEIFNNRKDVQCLIPCAIDQDPYFRMTRDVAPRIGFPKPALLHS
Frog        ACGFDVNKTFIFSDLEYMGKSSGFYQNVVKVQKHVTFNQVKGIFGFTDSDCIGKISFPAIQAAPSFSSSFPEIFKGRKDIQCLIPCAIDQDPYFRMTRDVAPRINYPKPALMHS
Fugu        ACGFDVNKTFIFSDLDYMGASPEFYRNVVKIEKHVTFNQVKGIFGFTDSDCIGKISFPAIQAAPSFSNSFPQIFGSRKDIQCLIPCAIDQDPYFRMTRDVAPRIGYPKPALLHS
Zebrafish   ACGFDVNKTFIFSDLEYMGASPAFYRNVVKVQKHVTFNQVKGIFGFTDSDCIGKISFPAIQAAPSFSNSFPQIFGDRKDVQCLIPCAIDQDPYFRMTRDVAPRIGYLKPALLHS

                                                                                                       418                              451
Human       TFFPALQGAQTKMSASDPNSSIFLTDTAKQIKTKVNKHAFSGGRDTIEEHRQFGGNCDVDVSFMYLTFFLEDDDKLEQIRKDYTSGAMLTGELKKALIEVLQPLIAEHQARRKE
Macaque     TFFPALQGAQTKMSASDPNSSIFLTDTAKQIKTKVNKHAFSGGRDTIEEHRQFGGNCDVDVSFMYLTFFLEDDDKLEQIRKDYTSGNMLTGELKKVLIEVLQPLIAEHQARRKE
Bovine      TFFPALQGAQTKMSASDPNSSIFLTDTAKQIKTKVNKHAFSGGRDTVEEHRQFGGNCDVDVSFMYLTFFLEDDDKLEQIRRDYTSGAMLTGELKKELIEVLQPLIAEHQARRKE
Chicken     VFFPALQGAQTKMSASDVNSSVFLNDTPKQIKTKINKHAFSGGRDTIEEHRKYGGNCDVDVSFMYLTFFLEDDDKLEQLKQAYTSGELLTGELKKVLIETLQPLIAAHQERRKQ
Snake       VFFPALQGAQTKMSASDPNSSIFLTDTPKQIKTKINKHAFSGGKDTVEEHRQYGGNCDVDVSYMYLTFFLEDDDRLEQIKQAYTSGALLTGELKKVLIETLQPLIAAHQQRRKQ
Frog        TFFPALQGAQTKMSASDPNSSIFLTDTAKQIKNKINKYAFSGGKDTIEEHRQFGGNWEVDVSYMYLTFFLEDDERLEQIKQDYSSGALLTGELKKILIETLQPLIAAHQERRKH
Fugu        TFFPALQGAQTKMSASDANSSIFLTDTPKQIKTKVNKHAFSGGKDTVEEHRKHGGNPDVDVSFMYLTFFLEDDEQLEKIRQDYASGALLTGELKKMLIDTLQPMIAQHQERRKQ
Zebrafish   TFFPALQGAQTKMSASDANSTIFLTDTPKQIKNKVNKHAFSGGKDTIEEHRKLGGDPDVDVSFMYLTFFLEDDEQLEKIRQDYSSGAMLTGELKKSLIDTLQPIIAEHQEKRKH

                               471
Human       VTDEIVKEFMTPRKLSFDFQ
Macaque     VTDEIAKQFMTPRELSFDFQ
Bovine      VTDEIVKEFMTPRKLSYDFQ
Chicken     ITDEVVKQFMTPRKLAFEF-
Snake       ITDEMVKEFMTPRKLAFDY-
Frog        ITEEAVKQFMMPRKLAFDF-
Fugu        VTDETVKQFMTPRPLNFKH-
Zebrafish   VTDDIVQQFMTPRKLHFSC-
```

Supplementary Figure S2. Sequence alignments among vertebrate TrpRS proteins. Multiple sequence alignment was performed by Clustal W with manual adjustments. Gaps in the sequences are indicated by dashes. Numbers above the sequences correspond to those of the residues of human TrpRS. Conserved crucial acidic (Glu or Asp) and basic (Arg or Lys) residues are highlighted in yellow.
